# Esketamine in persistent long COVID with predominant psychiatric manifestations: A case series

**DOI:** 10.1192/j.eurpsy.2025.2066

**Published:** 2025-08-26

**Authors:** I. Bozic, D. Ivkic, L. Reinfried, J. Donath, C. Schmidt, S. Graf, P. A. Handschuh, M. Dold, D. Winkler, A. Naderi-Heiden, N. Praschak-Rieder, D. Rujescu-Balcu, A. Weidenauer, L. Bartova

**Affiliations:** 1 Psychiatry and Psychotherapeutic Medicine; 2 Comprehensive Center for Clinical Neurosciences and Mental Health, Medical University of Vienna, Vienna, Austria

## Abstract

**Introduction:**

The COVID-19 pandemic has led to a significant number of patients presenting with post COVID-19 condition, commonly referred to as long COVID, which can affect any individual exposed to SARS-CoV-2, resulting in diminished quality of life, reduced productivity, increased healthcare expenditures, and broader economic implications. The most prevalent symptoms include neuropsychiatric manifestations such as fatigue, cognitive impairment, anxiety, and depression. Beneficial effects of Silexan, a herbal medicine derived from Lavandula angustifolia, were reported in long COVID patients with subsyndromal psychiatric symptoms (Bartova et al. Eur Neuropsychopharmacology 2023;70:47-48). However, research is lacking regarding psychopharmacotherapy in patients with persistent symptoms. Esketamine, noted for its modulation of NMDA receptors, has also demonstrated immunomodulatory effects, positioning it as a promising intervention for Long COVID (Johnston et al. Neuropsychopharmacology. 2024; 49(1): 23-40).

**Objectives:**

Our objective was to examine two patient cases to identify patterns, explore potential treatment options, and contribute insights to clinical practice in psychiatry.

**Methods:**

This case series reports the clinical histories, demographic information, diagnostic findings, and treatment details of two long COVID patients who were treated in analogy to the well-established guideline for treatment-resistant depression.

**Results:**

A 33-year-old female patient, who failed to respond to phytotherapy and conventional psychopharmacological treatments, including two trials of antidepressants and augmentation with an atypical antipsychotic agent received 10 intravenous esketamine treatments, administered at doses of up to 50 mg (0,86 mg/kg/hour). She experienced substantial clinical improvement without any adverse effects within 8 weeks. A 34-year-old non-responding female patient received 9 sessions of intranasal esketamine, targeting a dosage of 84 mg, resulting in complete remission without significant adverse effects within 6 weeks.

**Conclusions:**

There is an urgent need for effective and sustainable treatment options that address the debilitating neuropsychiatric symptoms of long COVID. This condition disproportionately affects young women, a group that is frequently underrepresented in research and insufficiently recognized in clinical practice. In this case series, we report on two female patients with severe physical and social impairment from long COVID, who showed significant clinical improvement following add-on esketamine administration.

**Disclosure of Interest:**

I. Bozic: None Declared, D. Ivkic: None Declared, L. Reinfried: None Declared, J. Donath: None Declared, C. Schmidt Grant / Research support from: Eli Lilly, S. Graf: None Declared, P. Handschuh: None Declared, M. Dold Grant / Research support from: Medizin Medien Austria, Janssen and Universimed, Consultant of: Medizin Medien Austria, Janssen and Universimed, D. Winkler Paid Instructor of: Angelini, Lundbeck, Medical Dialogue, and MedMedia Verlag, A. Naderi-Heiden: None Declared, N. Praschak-Rieder: None Declared, D. Rujescu-Balcu Grant / Research support from: Janssen and Lundbeck, Consultant of: Janssen and Rovi, Speakers bureau of: Janssen and Pharmagenetix, A. Weidenauer: None Declared, L. Bartova Grant / Research support from: Alpine Market Research, Angelini, Biogen, Diagnosia, Dialectica, EQT, Iqvia, Janssen, Johnson & Johnson, Lundbeck, Market Access Transformation, Medizin Medien Austria, Novartis, Schwabe and Universimed, Consultant of: Alpine Market Research, Angelini, Biogen, Diagnosia, Dialectica, EQT, Iqvia, Janssen, Johnson & Johnson, Lundbeck, Market Access Transformation, Medizin Medien Austria, Novartis, Schwabe and Universime

